# Novel optimized drug delivery systems for enhancing spinal cord injury repair in rats

**DOI:** 10.1080/10717544.2021.2009937

**Published:** 2021-12-02

**Authors:** Man Zhang, Yang Bai, Chang Xu, Jinti Lin, JiaKang Jin, Ankai Xu, Jia Nan Lou, Chao Qian, Wei Yu, Yulian Wu, Yiying Qi, Huimin Tao

**Affiliations:** a Department of Orthopedic Surgery, the Second Affiliated Hospital, Zhejiang University School of Medicine, Hangzhou City, PR China; b Orthopedics Research Institute of Zhejiang University, Hangzhou City, PR China; c Key Laboratory of Motor System Disease Research and Precision Therapy of Zhejiang Province, Hangzhou City, PR China; d The Second Affiliated Hospital, Zhejiang University School of Medicine, Hangzhou City, PR China

**Keywords:** Spinal cord injury, microsphere, control release, Laponite hydrogel, neuroprotection

## Abstract

Effective and accurate delivery of drugs to tissue with spinal cord injury (SCI) is the key to rehabilitating neurological deficits. Sustained-release microspheres (MS) have excellent degradability and can aid in the long-term release of drugs. However, the burst release phenomenon can cause unexpected side effects. Herein, we developed and optimized an injectable poly(lactic-co-glycolic acid) (PLGA) MS loaded with melatonin(Mel), which were mixed further with Laponite hydrogels (Lap/MS@Mel, a micro-gel compound) in order to reduce the burst release of MS. Thus, these MS were able to achieve stable and prolonged Mel release, as well as synergistic Lap hydrogel in order to repair neural function in SCI by in situ injection. In clinical practice, patients with SCI have complicated conditions and significant inter-individual differences, which means that a single route of administration does not meet actual clinical needs. Thus, the nanospheres are synthesized and subsequently coated with platelet membrane (PM) in order to form PM/MS@Mel (nano-PM compound) for sustained and precision-targeted delivery of Mel intravenously in the SCI. Notably, optimized microsphere delivery systems have improved Mel regulation polarization of spinal microglial/macrophages, which can reduce loss of biomaterials due to macrophage-induced immune response during implantation of spinal cord tissue. These two new delivery systems that are based on MS provide references for the clinical treatment of SCI, according to different requirements.

## Graphical Abstract

Schematic representation of repair of neurological deficits using Lap/MS@Mel and PM/MS@Mel nanoparticles in a rat model of spinal cord injury.

**Figure:**
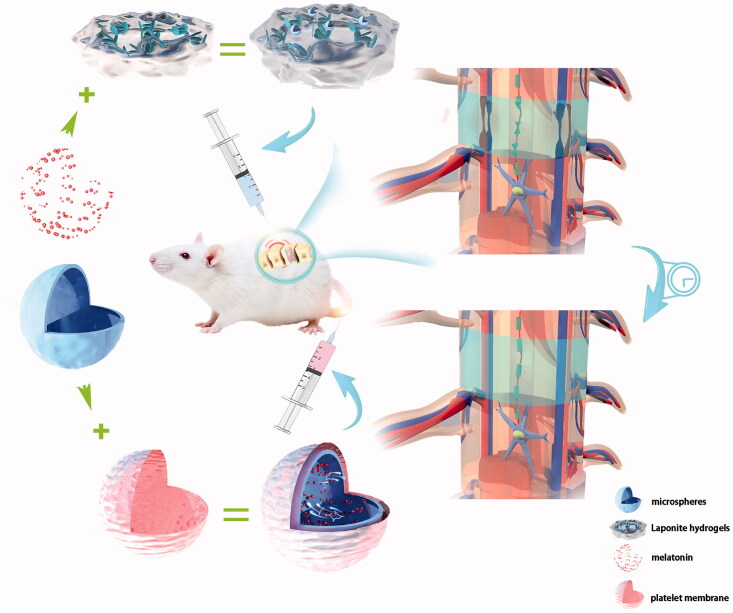

## Introduction

Spinal cord injury (SCI), which is one of the most serious injuries of the central nervous system, has a high rate of disability and serious complications, and has significant negative impact on daily life (McDonald & Sadowsky, 2002). Effective treatment at the early stages of SCI can have a great influence on the prognosis of SCI. However, due to short drug cycles and unclear targets, the current treatment strategy still has limitations. Pathological processes of SCI include primary and secondary injury (Ambrozaitis et al., 2006). Secondary injury is a long-term regulatory process at the cellular and molecular levels. In addition, its consequences are more serious than primary injury, which is also the main focus of current research on treatment strategies. The pathological mechanisms of secondary SCI are complex (Ahuja et al., 2017a,b; Karsy & Hawryluk, 2019), and include oxidative stress, mitochondrial dysfunction, nerve cell apoptosis, inflammatory response, lipid peroxidation and glutamate receptor overactivation. Among these, inflammation (Orr & Gensel, 2018) and oxidative stress (Jia et al., 2012) have important roles in the progression of SCI. In addition, due to ruptured and sheared blood vessels in SCI tissue (Yao et al., 2021), producing a high concentration of drugs at the local injury site, and to function continuously and effectively is quite difficult. Following spinal cord injury, Mel exerts neuroprotective effects by attenuating the inflammatory response (Yang et al., 2020) and oxidative stress response (Yuan et al., 2017). However, Mel has several disadvantages, including poor water solubility and easy decomposition. Thus, the purpose of this study is to establish a highly targeted drug delivery method to delivery drugs with definite efficacy (i.e. Mel) to intervene prior to secondary SCI and that can meet the needs of complex clinical applications.

Sustained-release microspheres (MS) are spherical entities that are formed by the dissolution or dispersion of drugs in the matrix of polymer materials. MS have several advantages, including an improvement in drug solubility, permeability and bioavailability (Hu et al., 2013). MS are often biodegradable and harmless to organisms, and are commonly composed of chitosan, methacrylate, gelatin, poly (lactic-co-glycolic acid, PLGA), as these materials can help achieve certain efficacy in the application of disease treatment. Studies have reported that methacrylate spheres loaded with diclofenac sodium, have excellent biocompatibility and can attenuate osteoarthritis (Yang et al., 2020). The IL4-loaded gelatin MS switched from a proinflammatory M1 macrophage into a pro-healing M2 phenotype, which efficiently resolved inflammation, and ultimately enhanced osteoblastic differentiation and bone regeneration (Hu et al., 2018). Furthermore, PLGA-MS loaded with growth factor sustained release system have been reported to promote sciatic nerve repair in rats (Zhang et al., 2017). Therefore, application of MS in SCI has been considered to be a potential treatment.

Hydrogels are hydrophilic polymers with a three-dimensional grid structure, which can absorb and retain a large amount of water or biological fluids. Based on their biocompatibility, biodegradability and good tolerance, hydrogels have been widely used in the field of drug targeted delivery and controlled release (Oliva et al., 2017). Laponite XLG (Na + 0.7[(Si8Mg5.5Li0.3)O20(OH)4] − 0.7) is a type of biocompatible hydrogel nanomaterial with a special structure, which includes a positively charged edge and negatively charged surface (Das et al., 2019). This nanomaterial is able to generate a stable nanoscale platelet dispersion and a large surface area in order to form a “House of Cards” structure when dispersed in solution due to electrostatic adsorption (Dávila & d’Ávila, 2017). Furthermore, this structure can be degraded into nontoxic products (Na+, Mg2+, Si(OH)4 and Li+); Na + and Mg2+ are beneficial to nerve cells (Tomás et al., 2018; Zhai et al., 2018). Based on these features, the nanomaterials are often used in the field of central nervous system repair and regenerative medicine. The Brimonidine-LAPONITE^®^ intravitreal formulation has been reported to have an ocular hypotensive and neuroprotective effect in a glaucoma animal model (Rodrigo et al., 2020). In addition, the laponite hydrogel bridge FGF4 has been shown to treat SCI (Wang et al., 2019). However, there are still a few studies that have evaluated whether laponite hydrogel can help promote stabilization of additional drug sustained-release biomaterials in the model of nerve damage.

Biological membrane coating is an effective tool for nanoparticle drug carriers in order to improve their biological properties (Zou et al., 2020). The membrane is nondestructive and extracted by a variety of physical and chemical methods, and then wrapped on the surface of inorganic or organic nanocarrier. Thus, they have similar biological functions to cells from the membrane. The use of cell membrane-coated nanodrugs that simulate source cells with a natural cell membrane of good biocompatibility, and the ability to interact in an *in vivo* microenvironment can identify and target source cells, extend its blood half-life, enhance accumulation in the target area, reduce immunogenicity, and minimize side effects (Luk & Zhang, 2015; Kroll et al., 2017; Qin et al., 2020). The sources of biomimetic materials include red blood cells, white blood cells, stem cells, tumor cells and platelets. Among these, platelets, which are an important cell type, are involved in the process of coagulation and hemostasis, innate immune response, and bacterial infection. Some studies in the treatment of cardiovascular atherosclerosis (Wei et al., 2018), rheumatoid arthritis (Jin et al., 2018), and cancer (Jiang et al., 2020) using a platelet membrane as a carrier of nanocoating have led to desirable outcomes. Furthermore, platelet membranes have the ability to naturally target the hemorrhage and inflammatory site, and do not need to rely upon passive targeting and active targeting by ligands and external stimulation. Thus, in view of the pathological characteristics of secondary SCI, application of platelet membrane is worth looking forward to.

Herein, given the clinical complexity of patients with SCI and the need for different routes of administration, we designed two novel injectable microsphere drug delivery systems. The Lap/MS@Mel drug delivery system has a drug-loading efficiency (DL%,7.2–9.1%) of MS that were loaded Mel mixed with the Laponite hydrogels, as the Laponite hydrogel can maintain bioactivity of Mel and prolonged and stabilize the MS to release Mel to the SCI tissue, thus synergistically repairing damaged nerves. The PM/MS@Mel is another delivery system that we have designed based on nanospheres for clinical treatment of SCI. The nanoscale MS cloud can pass through various narrow barriers in the blood system in order to reach site of injury. Importantly, MS that are subsequently coated with platelet membranes can increase stability, biocompatibility and targeted release of the microsphere sustained-release system in the blood. Multiple comprehensive evidences, which include functional, histological and morphological assessments were performed to evaluate the biological effect of Lap/MS@Mel gel and PM/MS@Mel. In addition, the novel sustained-release system helps Mel balance between the macrophage subsets that shift from the pro-inflammatory M1 phenotype to the anti-inflammatory M2 phenotype in order to reduce the loss of biological materials. Overall, the novel sustained-release system based on MS is able to validate a more precise and efficient delivery for melatonin, and promotes the effect on recovery of SCI.

## Materials and methods

### Lap/MS@Mel hydrogels and PM/MS@Mel nanoparticle fabrication

In order to prepare Lap/MS@Mel hydrogels, 10 mg of melatonin (Aladdin, Shanghai, China) and 100 mg of PLGA (molecular weight 40000; LA: GA =50:50, Aladdin, Shanghai, China) were dissolved in 3 mL of dichloromethane solution (National Pharmaceutical Group, Shanghai, China) in the oil phase. Then, they were mixed with 10 mL of deionized aqueous solution containing (2–4%, w/v) polyvinyl acetate. The emulsion was then prepared by phacoemulsification (400 ms/time, 8 times) under ice bath conditions. The emulsion was quickly added to 30 mL of aqueous solution, stirred overnight at 200–300 rpm, and the dichloromethane was volatilized. After, MS, loaded with Mel, were collected via centrifugation at 10,000 rpm, and then washed with distilled water three times, and freeze-dried for 24 h. Blank MS without drugs were then prepared using the same method. Then, 1.5 g of Laponite powder (Bick Chemical Co., Ltd, Germany) and loaded with MS@Mel was dissolved in 50 mL double distilled water by stirring for 2 h in order to form Lap/MS@Mel hydrogels.

For PM/MS@Mel nanoparticle, platelet membrane (PM) extraction, 10% Acid Citrate Dextrose (ACD, Solarbio, Beijing, China) anticoagulant was added to rat blood, and platelet-rich plasma (PRP) was collected after centrifugation at approximately 10,000 rpm for 20 min. The platelet precipitation was then collected after PRP was centrifuged at approximately 2000 rpm for 20 min. Then, platelet precipitation was resuspended in a red blood cell lysis buffer for 5 min at 4 °C, following centrifugation at approximately 2000 rpm for 20 min. After removing the supernatant, platelet precipitation was resuspended in the tyrode solution (Solarbio, Beijing, China) and frozen and thawed three times. The precipitate that was obtained after centrifugation was platelet membrane (PM). For MS@Mel nanoparticles synthesis, melatonin and poly(lactic-co-glycolic acid) (PLGA) were initially weighed and added to dichloromethane. After they were fully dissolved, they were added to the 3% bovine serum albumin (BSA, Solarbio, Beijing, China) solution. The mixture was sonicated for 5 min. Then, the above solution was added to 20 mL of saturated melatonin solution and stirred for 4 h. The MS@Mel nanoparticles were collected by centrifugation at 10,000 rpm for 20 min at 4 °C, and the supernatant was removed. The nanoparticles were then resuspended in deionized water by vortexing, and collected via centrifugation, again. The obtained platelet membrane and MS@Mel nanoparticles were then ultrasonicated for 30 min under the ultrasonic cleaning apparatus, and the obtained mixture was PM@MS@Mel.

### Characterizations

For encapsulation efficiency (EE), the sample (MS@Mel) was added to an Eppendorf (EP) tube, after weighing the tube. Next, the total weight of an EP tube was measured after lyophilization. The weight of the sample (MS@Mel) (WT) is equal to the total weight, minus the weight of the EP tube. Next, the sample was washed with distilled water, the supernatant was collected after centrifugation at 12,000 rpm, and then the amount of free Mel (WM) was detected using ultraviolet (absorbance value at 270 nm). The sediment (MS) was dissolved in dichloromethane containing deionized aqueous solution and stirred for 4 h, and the amount of encapsulated Mel (EM) was calculated by ultraviolet (absorbance value at, 270 nm). The encapsulation efficiency (EE) and drug loading efficiency (DL) was calculated according to the following formula: EE%=(EM/WM + EM)×100%.DL%=(EM/WT) ×100%. For Lap/MS@Mel hydrogels, EE%=69.3–71.5% and DL%=7.2–9.1%. For PM/MS@Mel nanoparticle, EE%=29.8–31.1% and DL%=2.7–3.5%.

For drug release experiment, the sample was put into 10 mL simulated body fluid (pH = 7.4), and the drug release rate was carried out under a constant temperature (37 °C ± 1 °C). For micro-gel compound, all release medium was changed at 1, 3, 5, 7, 14, 21, and 28 d, respectively, and the absorbance of the release medium was measured at the same time. For nano-PM compound, part of release medium was changed by the dialysis bag (Sbjbio Biotechnology Co., LTD, Nanjing, China) at 6, 12, 24, 48, 72, 96, 120, 144, and 168 h, respectively. The release amount was calculated, as per the standard curve equation. The zeta potentials of samples were investigated on a Zeta sizer (Malvern Instruments, UK), the absorption spectra were evaluated on a TU-1810 UV-V spectrophotometer and Fourier Transform Infrared (FTIR) Spectroscopy. Transmission electron microscopy (TEM) images were obtained using transmission electron microscope (TEM; Tecnai F20, FEI).

### Animals and spinal cord injury rat model construction

Sprague-Dawley (SD) rats were purchased from the Shanghai Laboratory Animal Center, Chinese Academy of Science (Shanghai, China). All animal experiments were conducted in compliance with guidelines, and followed a protocol that was approved by the Research Ethics Committee of Zhejiang University, China. In order to develop a spinal cord injury (SCI) model, animals were anesthetized with 2% (w/v) pentobarbital sodium (intraperitoneal injection; 40 mg/kg). The T9 lamina was removed, and then the T9 spinal cord was clamped using a 30 g vascular clamp (Oscar, China) for 1 min. Immediately, the Lap/MS hydrogel, a free Mel solution (20 mg), MS-containing 20 mg Mel or Lap/MS hydrogel containing 20 mg Mel, was orthotopically injected in order to cover the injured site. In addition, the PM/MS solution, a free Mel solution (5 mg/kg), MS containing Mel (5 mg/kg) or PM/MS containing Mel (5 mg/kg) was caudal vein that was injected with a time interval of seven days.

### Locomotion recovery assessment

The motor function of both hind limbs was analyzed on 1, 3, 7, 14 and 28 d after operation using the Basso Beattie Bresnahan (BBB) scale. The physiological changes, including range and number of joint movements, body balance, weight-bearing and coordination of the hind and forelimbs, were recorded ranging from 0 (complete paralysis) to 21 (normal locomotion).

### ELISA

The samples were measured for expression of tumor necrosis factor-alpha (TNF-α), interleukin-6 (IL-6), glutathione peroxidase (GPx), malondialdehyde (MDA) and superoxide dismutase (SOD) by ELISA kits (Boster Biological Technology co. Ltd, China).

### Western blotting

Proteins from tissues were extracted using the Radio Immunoprecipitation Assay (RIPA) lysis buffer. Then, 60 μg of protein was added per well, and separated on 4–20% gels. The protein was then transferred to the polyvinylidene fluoride (PVDF) membranes. Next, the PVDF membranes were blocked (5% fat-free milk) for one hour, and incubated overnight at 4 °C with following primary antibodies. These antibodies included c-caspase3 (1:1000, CST), Integrin α6 (1:1000, CST), CD41 (1:1000, CST), CD47 (1:1000, CST), CD62p (1:1000, CST), Histone H3 (1:1000, CST) and GAPDH (Cat: RT1210-1, Huabio). Next, the PVDF membranes were cultured with secondary antibodies at room temperature for one hour, and visualized by a ChemiDicTM XRS + Imaging System (BioRad Laboratories, Hercules, CA, USA).

### Immunofluorescence

Next, the samples were subjected to dewaxing, rehydration, dehydration, antigen repair, and then blocked by 5% bovine serum albumin for 30 min. These samples were incubated with primary antibodies, anti-cleaved caspase 3 (1:400), anti-Iba-1 (1:200), anti-Arginase-1 (1:200) or anti iNOS (1:100) at 4 °C overnight. This was followed by incubation with secondary antibodies for one hour. Next, all images were captured by a confocal laser microscope (Nikon, A1PLUS, Tokyo, Japan).

### Hematoxylin and eosin (H&E) staining

The tissue sections were stained with H&E and crystal violet, according to manufacturer's instructions. The images were captured by a Nikon ECLPSE 80i (Nikon, Tokyo, Japan).

### Statistical analysis

Data is presented as means ± SEM. One‑way ANOVA, followed by Tukey’s post hoc test, were used to determine differences among multiple groups. Repeated measurement two-way mixed ANOVA, followed by Tukey test, was utilized to detect differences between groups in BBB scores. *p*-Value <.05 was considered significant.

## Results

### Characteristics of Laponite and Lap/MS@Mel gels

**Figure 1.**
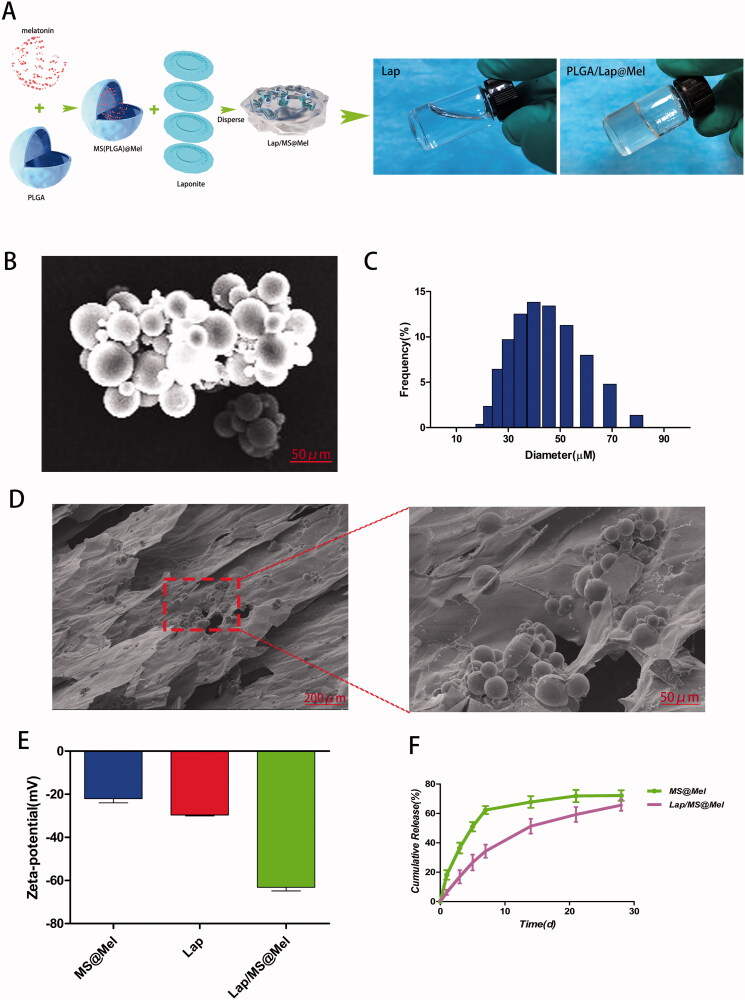
(A) Schematic illustration of the synthesis of Lap/MS@Mel. (B,D) SEM images of Lap/MS@Mel and MS@Mel. (C) The particle size of MS@Mel. (E) Zeta potential MS@Mel, Lap and Lap/MS@Mel. (F) Release of Mel from Lap/MS@Mel complexes.

A MS-based Laponite hydrogels sustained release system was synthesized (Figure 1(A)). The morphology of the Laponite and Lap/MS@Mel hydrogels was observed using a scanning electron microscope (SEM), as shown in Figure 1(B,D)), MS had a diameter of approximately 40 μm (Figure 1(C)) attached to the Lap hydrogen. In order to examine whether Mel and PLGA bind successfully, we measured the zeta potential and found that the zeta potential values of the MS@Mel, Laponite hydrogels and Lap/MS@Mel were −22, −29 and −63 mv (Figure 1(E)), respectively, which provides good evidence for the hypothesis that PLGA MS have an affinity for Lap hydrogels via electrostatic reaction. Drug release test results demonstrated that Lap/MS@Mel continuously released Mel for at least 28 days in vitro, however, a negligible amount of Mel was released from the MS@Mel after day 7 (Figure 1(F)).

### The characteristic of PM/MS@Mel

**Figure 2.**
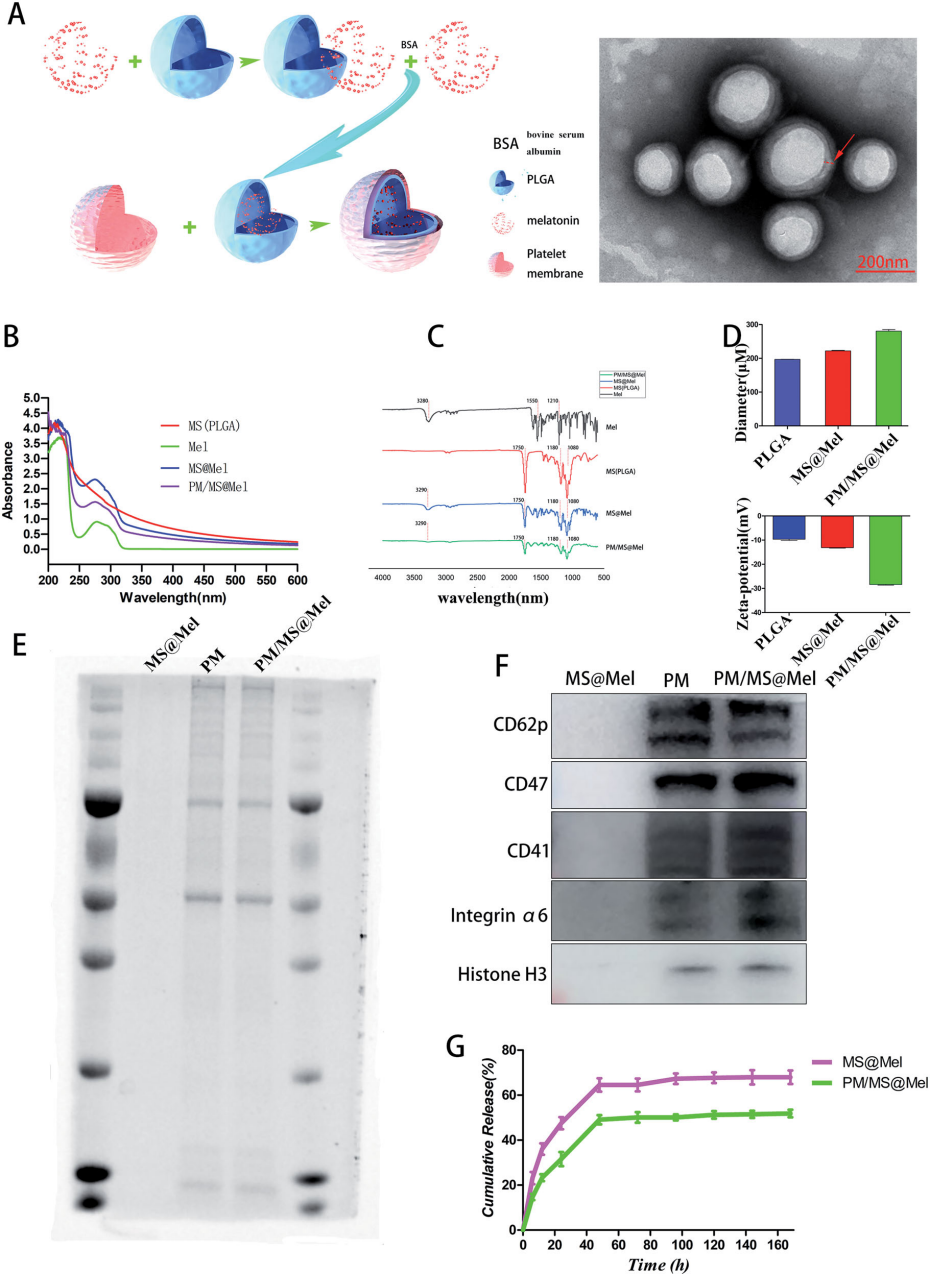
(A) TEM images of PM/MS@Mel nanoparticles. (B) The absorbance changes of nanoparticles at different wavelengths. (C) FT-IR spectra for Mel, PLGA, MS@Mel and PM/MS@Mel. (D) The particle size and zeta potential of PLGA, MS@Mel and PM/MS@Mel nanoparticles. (E) The Coomassie blue staining results of PLGA, MS@Mel and PM/MS@Mel nanoparticles. (F) Western blotting. (G) *In vitro* melatonin release from PM/MS@Mel nanoparticles.

As shown in Figure 2(A), a platelet membrane (PM) was coated outside the MS@Mel nanoparticles. The transmission electron microscope (TEM) demonstrated a thin biofilm wrap around the surface of the MS. The characteristic absorption peak of MS@Mel and PM/MS@Mel appear to be in the range of 250–300 nm wavelength (Figure 2(B)), which is consistent with the wavelength range of melatonin. The FT-IR results showed that (Figure 2(C)), for free Mel, the characteristic absorption peak of N-H stretching vibration was visible at 3280 cm^−1^. Furthermore, the characteristic absorption peaks of benzene ring C–H stretching vibration were observed at 1550 and 1210 cm^−1^. For PLGA, there is an absorption peak generated by C=O stretching vibration at 1750 cm^−1^. In addition, at 2950 cm^−1^, the absorption peak was saturated with C=H stretching vibration. In the encapsulated nanoparticles (MS@Mel,PM/MS@Mel), all the above peaks are present, then the encapsulation was successful. The diameter of MS, MS@Mel and PM/MS@Mel were 196.8, 222.1 and 280.7 nm (Figure 2(D)), respectively. Hence, this result indicates that melatonin was favorably wrapped by PLGA MS. In order to further test whether MS@Mel nanoparticles have been favorably wrapped by PM, the protein mark on PM was investigated. Coomassie blue staining (Figure 2(E)) and western blotting (Figure 2(F)) results demonstrated that the unique platelet membrane protein mark (Integrin α6, CD41, CD47, CD62p) and nuclear protein marker Histone H3 are detected in the group of PM and PM@MS, however, these indicators were not found in the MS group. Compared to MS@Mel group of nanoparticles, the absolute value of size and zeta potential of nanoparticles in PM/MS@Mel group were slightly increased (Figure 2(D)). The drug release test results demonstrated that PM/MS@Mel continuously released the Mel for at least 7 d *in vitro* (Figure 2(G)).

### The drug delivery system improved pathology and motor function after SCI

**Figure 3.**
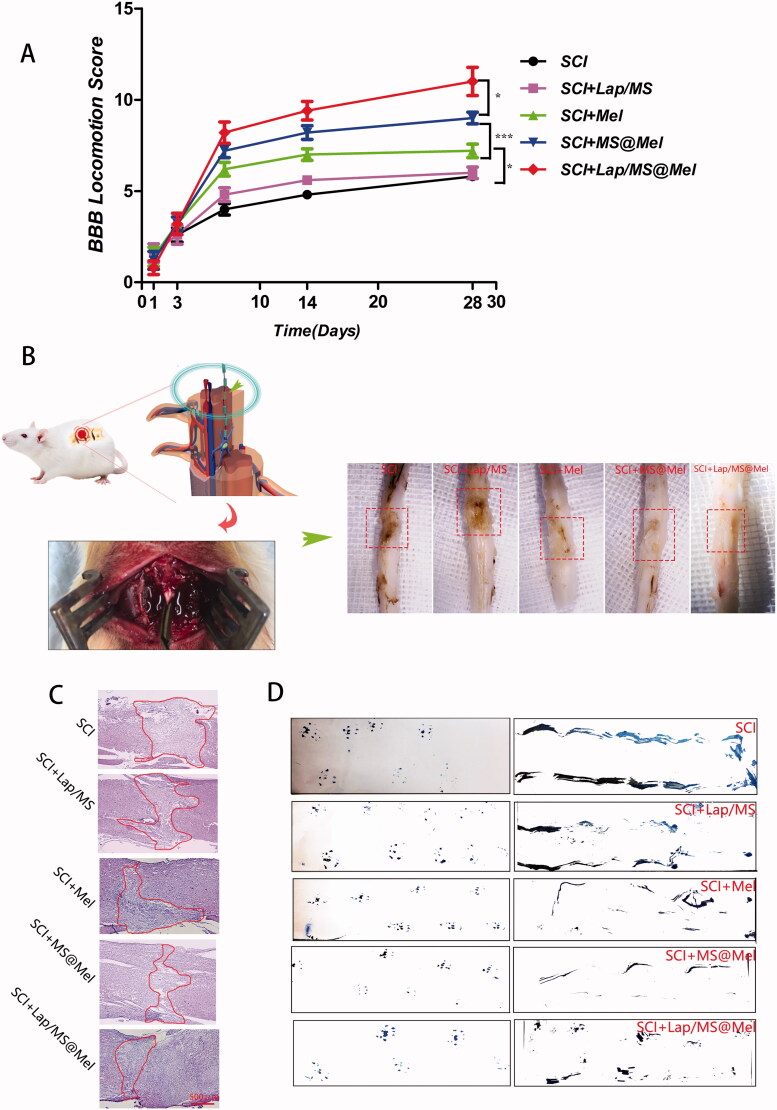
(A) Basso Beattie Bresnahan (BBB) score across different groups. (B) Schematic diagram of spinal cord injury in rats and images of harvested spinal cord tissue across different groups.(C) H&E staining of spinal cord tissue across different groups. (D) Footprint analyses. (*n* = 5 per group; *p < .05 versus SCI group; ***p < .001 or **p < .01 versus SCI + Mel group; *p < .05 versus SCI + MS@Mel group).

In order to detect neurological deficits in rats, the Basso Beattie Bresnahan (BBB) score was performed within four weeks after surgery. The BBB scores of the Lap/MS@Mel group were found to be significantly higher than those of the Mel and MS@Mel group for all time points from two weeks post-surgery (Figure 3(A)). The H&E staining and physical photos of spinal cord tissue demonstrated that severe injury was observed on the injured site of the spinal cord at day 28 after SCI. The hierarchy of the relative lesion area is as follows: SCI > Lap/MS > free Mel > MS@Mel > Lap/MS@Mel (Figure 3(B,C)). The footprint test demonstrated that the rats in Lap/MS@Mel group presented a coordinated and consistent posterior limb footprint at day 21 after SCI. Meanwhile, compared to the SCI group, the Lap/MS, MS@Mel and free Mel groups demonstrated that the width of blue ink streaks was still increased in these groups (Figure 3(D)).

**Figure 4.**
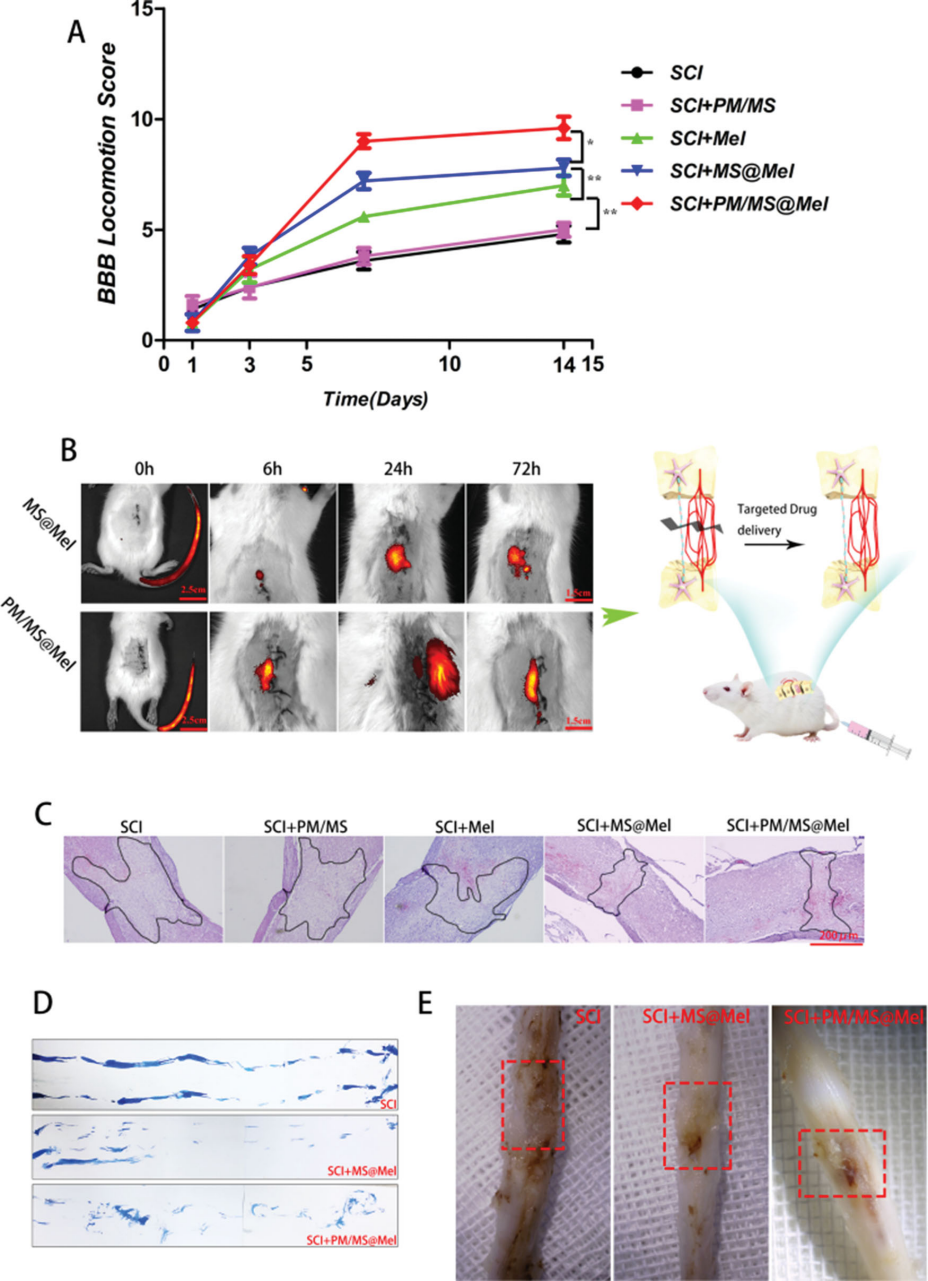
(A) Basso Beattie Bresnahan (BBB) score across different groups. (B) Schematic diagram of spinal cord injury in rats and *in vivo* spectrum imaging system (IVIS) images of spinal cord injected with MS-conjugated Cy7.5 after spinal cord injury (SCI).(C) H&E staining of spinal cord tissue across different groups. (D) Footprint analyses. (E) Images of harvested spinal cord tissue across different groups (*n* = 5 per group; **p < .01 versus SCI group; **p < .01 versus Mel group; *p < .05 versus SCI + PM/MS@Mel group).

Being encouraged by the nerve repair efficacy of the micro-gel compound via the in situ injection in the SCI rat model, we next evaluated the efficacy of another common clinical administration, intravenous administration (nano-PM compound), for nerve repair. As presented in Figure 4(A)), the BBB score in the PM/MS@Mel group was higher than that of free Mel, MS@Mel and the PM/MS group after 7–28 days post-surgery. We demonstrated that Mel MS have neurorepair function in a rat model of SCI. Thus, we were able to evaluate the ability of nanoparticles to target spinal cord injury tissue in an intravenous microsphere experiment. As illustrated in Figure 4(B) by MS-conjugated Cy7.5 (MS-Cy7.5), the fluorescence intensity of PM/MS@Mel group was much stronger than that of the group MS@Mel, and the fluorescence intensity in the spinal cord gradually decreased over time. The H&E staining (Figure 4(C)), footprint test (Figure 4(D)) and physical photos (Figure 4(E)) of spinal cord tissue showed that the platelet coated drug delivery MS had an improved ability to repair nerve function than the uncoated drug delivery MS.

### The drug delivery system restrained SCI-induced apoptosis, oxidative stress and inflammatory response

**Figure 5.**
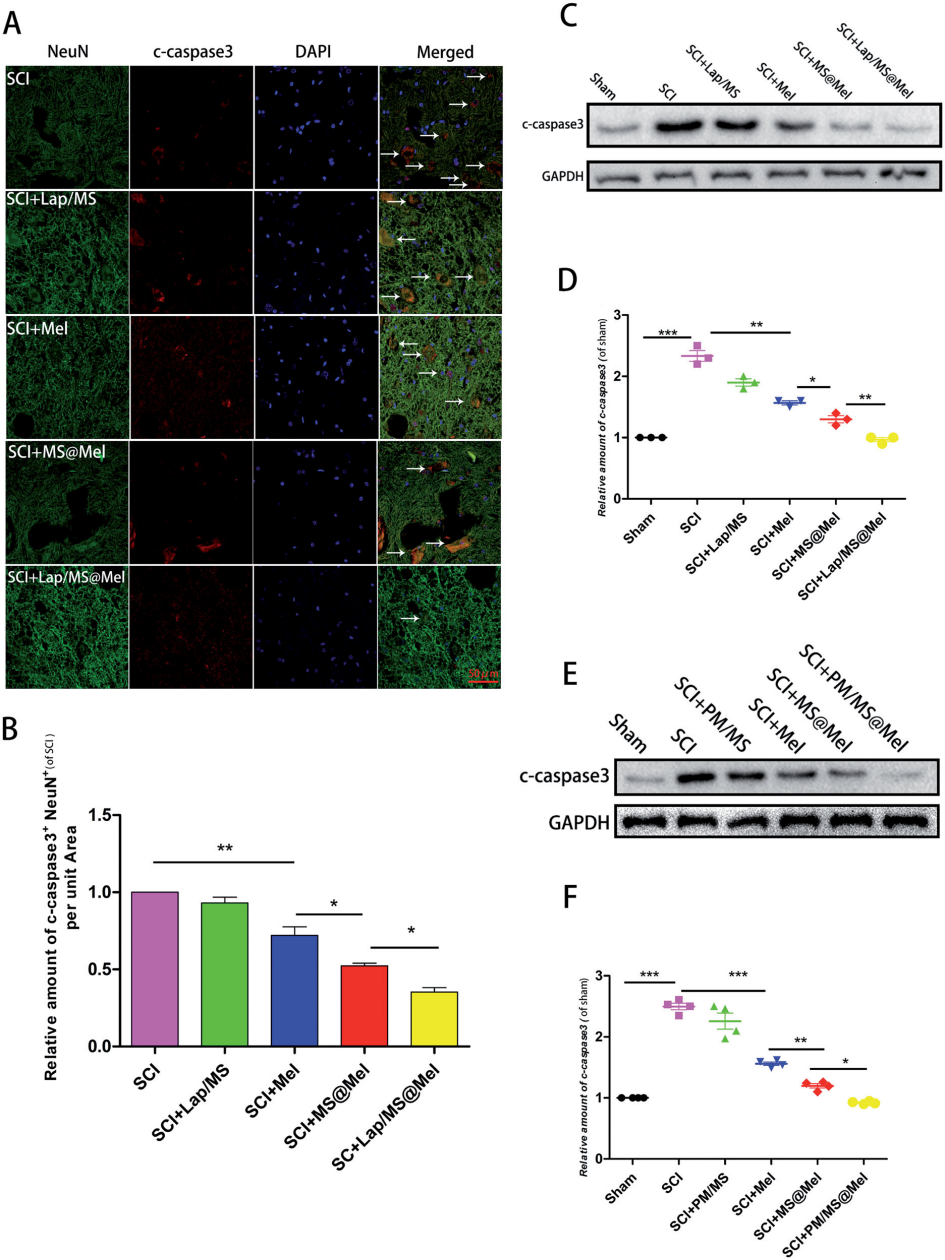
(A,B) Immunofluorescence and intensity co-staining of NeuN (green) and c-caspase3 (red) of the respective groups.(C-F) The protein expression and densitometric quantification of c-caspase3 across the respective groups (*n* = 5 per group).

**Figure 6.**
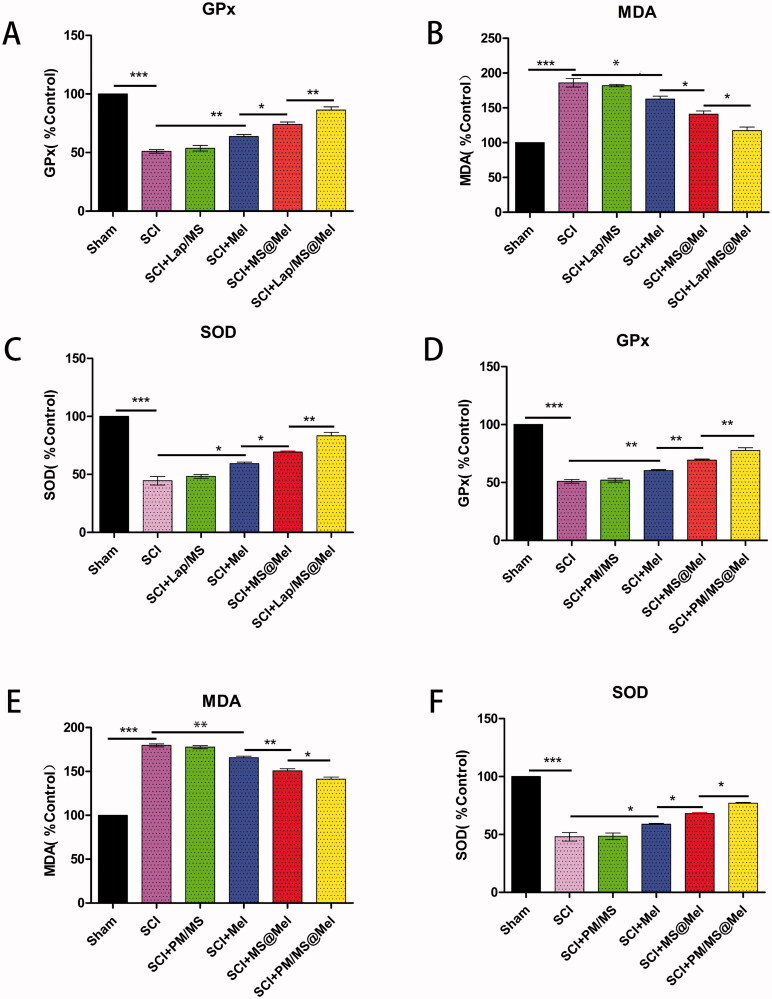
(A–F) The SOD, MDA and GPx levels of spinal cord tissue across different groups (*n* = 5 per group).

In order to further verify the inhibitory effect of the drug delivery system promoting Mel in inhibiting apoptosis of spinal cord tissue after SCI, we examined the expression of active-caspase3 proteins in the spinal cord tissue. For micro-gel compound, we discovered that the hierarchy of active-caspase3 is as follows: SCI > Lap/MS > free Mel > MS@Mel > Lap/MS@Mel (Figure 5(A–D)). The effect of Mel on inflammation and oxidative stress was previously demonstrated by numerous studies (Arioz et al., 2019; Rehman et al., 2019; Jauhari et al., 2020). In order to examine the effectiveness of drug delivery systems, we utilized the corresponding kits to measure the spinal cord tissue levels of malondialdehyde (MDA), glutathione peroxidase (GPx) and superoxide dismutase (SOD). As shown in Figure 6(A–C), the Lap/MS@Mel gels were able to inhibiting oxidative stress response in a rat model of SCI. Furthermore, the hierarchy of MDA was as follows: SCI > Lap/MS > free Mel > MS@Mel > Lap/MS@Mel. Meanwhile, the hierarchy of GPx and SOD was as follows: SCI < Lap/MS < free Mel < MS@Mel < Lap/MS@Mel.

For nano-PM compound, the expression of active-caspase3 proteins (Figure 5(E,F)) and expression of MDA, GPx and SOD in the spinal cord tissue (Figure 6(D–E)) had similar results to the micro-gel compound.

### The drug delivery system influenced the macrophage/microglia M1-M2 polarization balance after SCI

**Figure 7.**
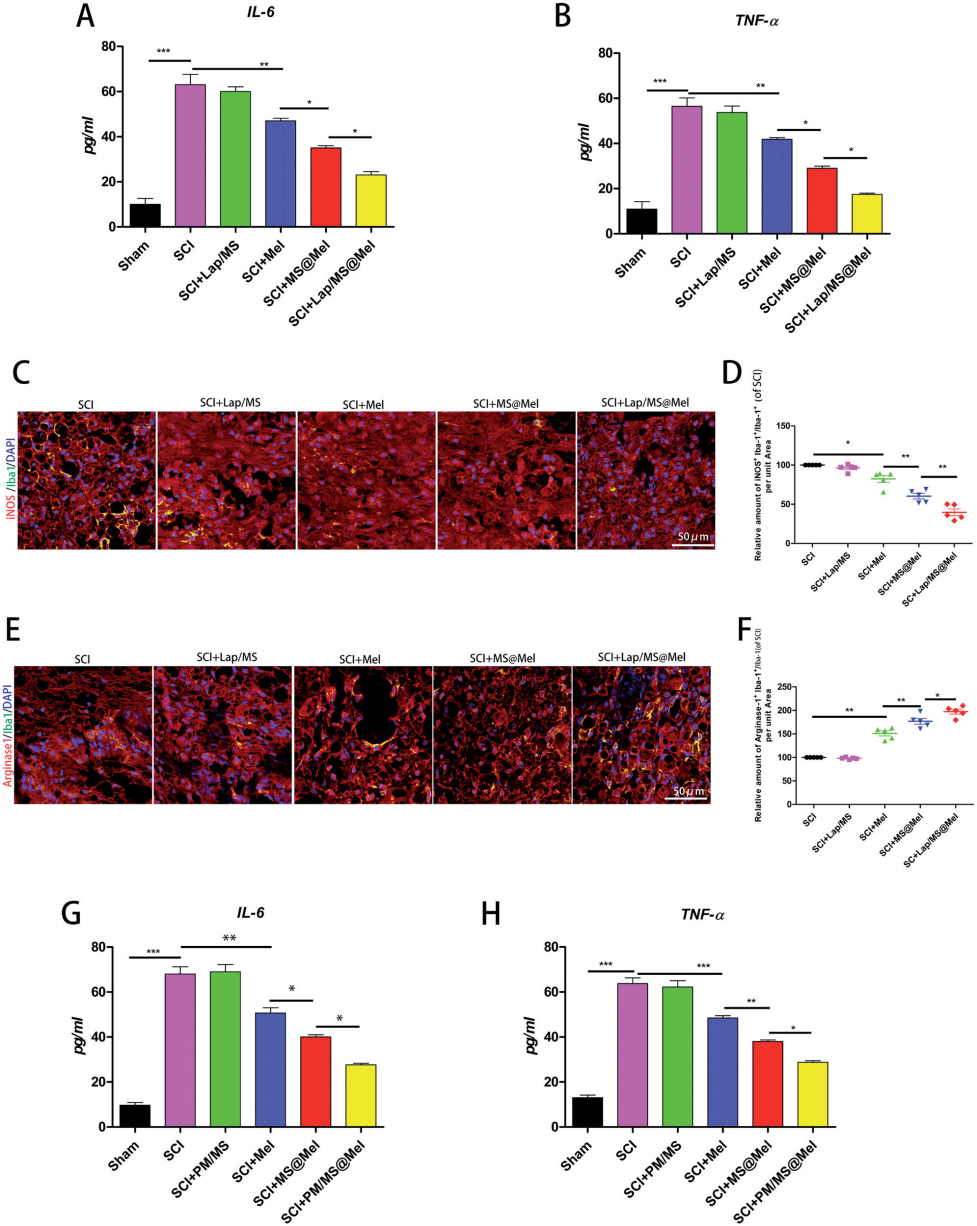
(A,B,G,H) The ELISA analysis of IL-6 and TNF-α in the spinal cord tissue of respective groups.(C,D) Immunofluorescence and intensity co-staining of Iba-1 (green) and iNOS (red) of the respective groups. (E,F) Immunofluorescence and intensity co-staining of Iba-1 (green) and Arginase1 (red) of the respective groups. (*n* = 5 per group).

As shown in Figure 7(A,B,G,H), the Lap/MS@Mel gels were able to reduce secretion of inflammatory factors in a rat model of SCI. Furthermore, the hierarchy of IL-6 and TNF-α was as follows: SCI > Lap/MS > free Mel > MS@Mel > Lap/MS@Mel. We demonstrated that the optimized MS promote melatonin anti-inflammatory effects in SCI model rats. Furthermore, macrophages/microglia phenotypic transition plays important roles in the inflammatory response toward SCI. Thus, we speculated that the effect of optimized MS in melatonin is likely by promoting melatonin to inhibit macrophages/microglia polarization to the M1 phenotype. In order to determine the effect of optimized MS on M1 polarization, we examined markers of M1 macrophages/microglia (iNOS) in spinal cord tissue at seven days after injury. Our results showed that iNOS levels were decreased in the Lap/MS@Mel group, and the hierarchy of iNOS was as follows: SCI > =Lap/MS > free Mel > MS@Mel > Lap/MS@Mel (Figure 7(C,D)). Furthermore, we examined the effect of optimized MS on M2 polarization. The data showed that the optimized melatonin MS increased expression of Arginase1 in spinal cord tissue at seven days after injury. Furthermore, the hierarchy of Arginase1 is as follows: SCI< =Lap/MS < free Mel < MS@Mel < Lap/MS@Mel (Figure 7(E,F)), these results showed that the Microsphere drug delivery system promotes functioning of melatonin by regulating macrophages/microglia M1-M2 polarization after SCI.

## Discussion

To date, the clinical treatment for SCI includes drug therapy combined with physical therapy (Rehman et al., 2019), such as calcium channel antagonists, hormones, and naloxone combined with hyperbaric oxygen therapy, which were used to reduce or eliminate secondary pathological reactions in the acute phase of injury and to protect the remaining axons and neurons from secondary injury. Furthermore, surgical treatment eliminates physical damage and promotes regeneration and repair of nerve tissue during a chronic period of injury. Although part of the damaged neurons are able to regenerate through these above treatment strategies, the sensory and motor abilities of patients with SCI are in the process of continuous injury progression due to persistence of secondary injury. Furthermore, the functional defects and obstacles caused by trauma have not yet had a fundamental breakthrough. Melatonin plays a vital role in human organs, as it regulates many essential pathological and physiological functions. Studies have confirmed that melatonin fastens the recovery of sciatic and hippocampal (Li et al., 2018) nerve injury (Rateb et al., 2017) in rats by inhibiting inflammatory cytokine secretion and oxidative stress. However, due to differences of water-solubility of melatonin, there is a lack of specificity and stability in body distribution. After melatonin enters the body, it is vulnerable to protease hydrolysis, which causes the oral, intravenous injection or in situ bioavailability to be low. In addition, due to the existence of blood spinal cord barrier – it can also influence the treatment effect. In order to overcome this disadvantage, more doses or repeated doses were selected during treatment, which can consequently result in unexpected damage to the human body. Direct or indirect infusion methods, such as melatonin intrathecal infusion, can effectively improve drug concentration in the spinal cord. However, the drug maintenance effect is short, the injury is large, the operation is complicated, and it is easy to cause an infection or aggravate spinal cord injury. For these above reasons, the clinical application of melatonin in the treatment of SCI remains limited.

As an effective method of drug delivery, drug-loaded sustained-release MS have been reported to improve efficacy of drug repair for peripheral nerve injury (Zhuang et al., 2016; Rao et al., 2019). In addition, MS have the potential to prolong retention time of melatonin in the nasal mucosa, improve bioavailability, as well as the therapeutic effect (Nižić et al., 2020). Furthermore, studies have shown that melatonin-loaded PLGA MS can help improve efficacy of glaucoma treatment (Arranz-Romera et al., 2019). However, the burst release associated with MS reduces efficacy of MS, and increases risk of unpredictable side effects in nerve repair and tissue regeneration engineering (Rambhia & Ma, 2015; Dong et al., 2020). Early works have utilized composite hydrogen–microsphere delivery systems in order to reduce the burst release, and improve regeneration of various tissues (Elisseeff et al., 2001; Dyondi et al., 2013; Karam et al., 2014). Therefore, we speculate that the hydrogel-bound MS can help achieve stable microsphere degradation and release of drugs, which can help avoid the phenomenon of peak and valley of blood drug concentration caused by microsphere burst release, reduce the dose of administration during treatment cycle, and improve the bioavailability of drugs and patient compliance. Laponite hydrogel is often utilized in tissue regeneration engineering due to its unique properties. One study highlighted the great potential of laponite-enhanced hydrogel MS in vascularized dental pulp regeneration (Zhang et al., 2020). Our results show that Lap hydrogels reduce the initial burst release of MS, and compared to MS@Mel, Lap/MS@Mel presents a more stable sustained release and better neural recovery ability of SCI via an *in situ* injection. After SCI, macrophages rapidly polarize into M1 macrophages and release inflammatory cytokine aggravated inflammation, inhibit axon regeneration, promote lipid oxidation and degradation, affect cell membrane fluidity and permeability, and resulting in re-injury of neurons and glial cells (David & Kroner, 2011; Milich et al., 2019). In addition, polarization of macrophages is recruited to the surface of the biomaterial after implantation. Then, a series of inflammatory cytokines is secreted, which may lead to degradation and failure of the biomaterial. Regulation of this type of cell has become an urgent problem. Our data shows that the MS-based sustained release system has the ability to enhance Mel regulation in the transformation of macrophages/microglia from an M1 phenotype to an M2 phenotype in SCI tissue.

Furthermore, we demonstrated the effectiveness of *in situ* Lap/MS@Mel gel system for SCI repair. However, due to *in situ* device injury and post-injection volume effect, improper operation will inevitably cause additional SCI. For the clinical complexity of patients with SCI, some patients are not suitable for an *in-situ* injection. Therefore, there should be other options for SCI administration. Besides, MS injected intravenously become consumed by macrophages of the reticuloendothelial system, and gets blocked by the blood-spinal cord barrier, which is difficult to pass through various narrow spaces in the blood system, and cannot effectively reach the SCI tissue. It is a common strategy to modify the surface of MS and alter the charge of the MS, as it can help increase the affinity of the MS to a specific target, and prolong the retention time of the drug *in vivo*, leading to enhanced efficacy of the drug. Previous studies have confirmed that the intravenous injection of nanosphere-delivering drugs has the effect of promoting optic nerve regeneration (Robinson et al., 2011). Furthermore, nanoparticle modified by platelet membrane cloaking has reduced cellular uptake by macrophage-like cells, as well as improved disease-targeted delivery of nanoparticles (Hu et al., 2015). Based on these above studies, we designed the PM/MS@Mel nano sustained release system and applied it to treatment of SCI for the first time. Our experimental results demonstrate that nanoscale MS can smoothly reach the injured site via various barriers and gaps in the blood system, and play the role of nerve repair. Meanwhile, compared to MS@Mel, PM/MS@Mel increases biocompatibility of MS and precision of delivery by taking advantage of the targeting characteristics of the platelet membrane.

## Conclusion

In conclusion, this study caters to the clinical complexity of patients with SCI, as we designed and synthesized two novel optimized drug delivery MS. The Lap/MS@Mel with high loading efficiency PLGA MS mixed with the Laponite hydrogel was found to facilitate and prolong melatonin delivery to the damaged spinal cord via an *in situ* injection *in vivo*. For another common drug delivery approach in the clinical treatment of SCI, we synthesized a PM/MS@Mel nanometer sustained-release system which uses nanoscale MS loaded with melatonin in order to avoid being blocked by various barriers and membrane gaps in the blood system. Meanwhile, the biomimetic platelet membrane encapsulated MS increase biocompatibility of the MS to prevent them from being engulfed by macrophages in the blood system and precision of delivery. The Lap/MS@Mel gel and PM/MS@Mel exerted neuroprotective effects and restrained oxidative stress and inflammatory reactions. Importantly, novel optimized drug delivery MS enhanced Mel-inhibition of macrophages/microglia polarization to the M1 phenotype and thus, prevented the biomaterial from being destroyed. The neuroprotection benefits of the two deliveries system are in line with clinical treatment strategy, have enormous potential, and are a clinically feasible therapeutic approach for patients suffering from SCI.

## References

[CIT0001] Ahuja CS, Nori S, Tetreault L, et al. (2017a). Traumatic spinal cord injury-repair and regeneration. Neurosurgery 80:S9–S22. 28350947 10.1093/neuros/nyw080

[CIT0002] Ahuja CS, Wilson JR, Nori S, et al. (2017b). Traumatic spinal cord injury. Nat Rev Dis Primers 3:17018. 28447605 10.1038/nrdp.2017.18

[CIT0003] Ambrozaitis KV, Kontautas E, Spakauskas B, Vaitkaitis D. (2006). Pathophysiology of acute spinal cord injury. Medicina (Kaunas, Lithuania) 42:255–61. 16607070

[CIT0004] Arioz BI, Tastan B, Tarakcioglu E, et al. (2019). Melatonin attenuates LPS-induced acute depressive-like behaviors and microglial NLRP3 inflammasome activation through the SIRT1/Nrf2 PATHWAY. Front Immunol 10:1511. 31327964 10.3389/fimmu.2019.01511 PMC6615259

[CIT0005] Arranz-Romera A, Davis BM, Bravo-Osuna I, et al. (2019). Simultaneous co-delivery of neuroprotective drugs from multi-loaded PLGA microspheres for the treatment of glaucoma. J Control Release 297:26–38. 30664980 10.1016/j.jconrel.2019.01.012

[CIT0006] David S, Kroner A. (2011). Repertoire of microglial and macrophage responses after spinal cord injury. Nat Rev Neurosci 12:388–99. 21673720 10.1038/nrn3053

[CIT0007] Dávila JL, d’Ávila MA. (2017). Laponite as a rheology modifier of alginate solutions: physical gelation and aging evolution. Carbohydr Polym 157:1–8. 27987800 10.1016/j.carbpol.2016.09.057

[CIT0008] Dong N, Zhu C, Jiang J, et al. (2020). Development of composite PLGA microspheres containing exenatide-encapsulated lecithin nanoparticles for sustained drug release. Asian J Pharm Sci 15:347–55., 32636952 10.1016/j.ajps.2019.01.002 PMC7327764

[CIT0009] Dyondi D, Webster TJ, Banerjee R. (2013). A nanoparticulate injectable hydrogel as a tissue engineering scaffold for multiple growth factor delivery for bone regeneration. Int J Nanomed 8:47–59. 10.2147/IJN.S37953 PMC3534298 23293519

[CIT0010] Elisseeff J, McIntosh W, Fu K, et al. (2001). Controlled-release of IGF-I and TGF-beta1 in a photopolymerizing hydrogel for cartilage tissue engineering. J Orthop Res 19:1098–104. 11781011 10.1016/S0736-0266(01)00054-7

[CIT0011] Hu CM, Fang RH, Wang KC, et al. (2015). Nanoparticle biointerfacing by platelet membrane cloaking. Nature 526:118–21. 26374997 10.1038/nature15373 PMC4871317

[CIT0012] Hu L, Zhang H, Song W. (2013). An overview of preparation and evaluation sustained-release injectable microspheres. J Microencapsul 30:369–82. 23140260 10.3109/02652048.2012.742158

[CIT0013] Hu Z, Ma C, Rong X, et al. (2018). Immunomodulatory ECM-like microspheres for accelerated bone regeneration in diabetes mellitus. ACS Appl Mater Interfaces 10:2377–90. 29280610 10.1021/acsami.7b18458 PMC6437671

[CIT0014] Jauhari A, Baranov SV, Suofu Y, et al. (2020). Melatonin inhibits cytosolic mitochondrial DNA-induced neuroinflammatory signaling in accelerated aging and neurodegeneration. J Clin Invest 130:3124–36. 32182222 10.1172/JCI135026 PMC7260019

[CIT0015] Jia Z, Zhu H, Li J, et al. (2012). Oxidative stress in spinal cord injury and antioxidant-based intervention. Spinal Cord 50:264–74., 21987065 10.1038/sc.2011.111

[CIT0016] Jiang Q, Wang K, Zhang X, et al. (2020). Platelet membrane-camouflaged magnetic nanoparticles for ferroptosis-enhanced cancer immunotherapy. Small 16:e2001704. 32338436 10.1002/smll.202001704

[CIT0017] Jin K, Luo Z, Zhang B, Pang Z. (2018). Biomimetic nanoparticles for inflammation targeting. Acta Pharm Sin B 8:23–33. 29872620 10.1016/j.apsb.2017.12.002 PMC5985691

[CIT0018] Karam JP, Muscari C, Sindji L, et al. (2014). Pharmacologically active microcarriers associated with thermosensitive hydrogel as a growth factor releasing biomimetic 3D scaffold for cardiac tissue-engineering. J Control Release 192:82–94. 24998940 10.1016/j.jconrel.2014.06.052

[CIT0019] Karsy M, Hawryluk G. (2019). Modern medical management of spinal cord injury. Curr Neurol Neurosci Rep 19:65. 31363857 10.1007/s11910-019-0984-1

[CIT0020] Kroll AV, Fang RH, Zhang L. (2017). Biointerfacing and applications of cell membrane-coated nanoparticles. Bioconjug Chem 28:23–32. 27798829 10.1021/acs.bioconjchem.6b00569 PMC5471317

[CIT0021] Li B, Feng XJ, Hu XY, et al. (2018). Effect of melatonin on attenuating the isoflurane-induced oxidative damage is related to PKCα/Nrf2 signaling pathway in developing rats. Brain Res Bull 143:9–18. 30278199 10.1016/j.brainresbull.2018.09.018

[CIT0022] Luk BT, Zhang L. (2015). Cell membrane-camouflaged nanoparticles for drug delivery. J Control Release 220:600–7. 26210440 10.1016/j.jconrel.2015.07.019 PMC4688192

[CIT0023] McDonald JW, Sadowsky C. (2002). Spinal-cord injury. Lancet (London, England) 359:417–25. 10.1016/S0140-6736(02)07603-1 11844532

[CIT0024] Milich LM, Ryan CB, Lee JK. (2019). The origin, fate, and contribution of macrophages to spinal cord injury pathology. Acta Neuropathol 137:785–97. 30929040 10.1007/s00401-019-01992-3 PMC6510275

[CIT0025] Nižić L, Potaś J, Winnicka K, et al. (2020). Development, characterisation and nasal deposition of melatonin-loaded pectin/hypromellose microspheres. Eur J Pharm Sci 141:105115. 31654755 10.1016/j.ejps.2019.105115

[CIT0026] Oliva N, Conde J, Wang K, Artzi N. (2017). Designing hydrogels for on-demand therapy. Acc Chem Res 50:669–79. 28301139 10.1021/acs.accounts.6b00536 PMC6527116

[CIT0027] Orr MB, Gensel JC. (2018). Spinal cord injury scarring and inflammation: therapies targeting glial and inflammatory responses. Neurotherapeutics 15:541–53. 29717413 10.1007/s13311-018-0631-6 PMC6095779

[CIT0028] Qin M, Du G, Sun X. (2020). Biomimetic cell-derived nanocarriers for modulating immune responses. Biomater Sci 8:530–43. 31750453 10.1039/c9bm01444f

[CIT0029] Rambhia KJ, Ma PX. (2015). Controlled drug release for tissue engineering. J Control Release 219:119–28. 26325405 10.1016/j.jconrel.2015.08.049 PMC4656104

[CIT0030] Rao F, Yuan Z, Zhang D, et al. (2019). Small-molecule SB216763-loaded microspheres repair peripheral nerve injury in small gap tubulization. Front Neurosci 13:489. 31156373 10.3389/fnins.2019.00489 PMC6530511

[CIT0031] Rateb EE, Amin SN, El-Tablawy N, et al. (2017). Effect of melatonin supplemented at the light or dark period on recovery of sciatic nerve injury in rats. EXCLI J 16:138–50., 28435433 10.17179/excli2016-763 PMC5379119

[CIT0032] Rehman SU, Ikram M, Ullah N, et al. (2019). Neurological enhancement effects of melatonin against brain injury-induced oxidative stress, neuroinflammation, and neurodegeneration via AMPK/CREB signaling. Cells 8:760. 10.3390/cells8070760 PMC6678342 31330909

[CIT0033] Robinson R, Viviano SR, Criscione JM, et al. (2011). Nanospheres delivering the EGFR TKI AG1478 promote optic nerve regeneration: the role of size for intraocular drug delivery. ACS Nano 5:4392–400., 21619059 10.1021/nn103146p PMC3136352

[CIT0034] Rodrigo MJ, Cardiel MJ, Fraile JM, et al. (2020). Brimonidine-LAPONITE® intravitreal formulation has an ocular hypotensive and neuroprotective effect throughout 6 months of follow-up in a glaucoma animal model. Biomater Sci 8:6246–60. 33016285 10.1039/d0bm01013h

[CIT0035] Das Neelam SS, Hussain K, Singh S, et al. (2019). Laponite-based nanomaterials for biomedical applications: a review. Curr Pharm Des 25:424–43. 30947654 10.2174/1381612825666190402165845

[CIT0036] Tomás H, Alves CS, Rodrigues J. (2018). Laponite®: a key nanoplatform for biomedical applications? Nanomedicine 14:2407–20. 28552649 10.1016/j.nano.2017.04.016

[CIT0037] Wang C, Gong Z, Huang X, et al. (2019). An injectable heparin-Laponite hydrogel bridge FGF4 for spinal cord injury by stabilizing microtubule and improving mitochondrial function. Theranostics 9:7016–32. 31660084 10.7150/thno.37601 PMC6815951

[CIT0038] Wei X, Ying M, Dehaini D, et al. (2018). Nanoparticle functionalization with platelet membrane enables multifactored biological targeting and detection of atherosclerosis. ACS Nano 12:109–16. 29216423 10.1021/acsnano.7b07720 PMC5859122

[CIT0039] Yang J, Han Y, Lin J, et al. (2020). Ball-bearing-inspired polyampholyte-modified microspheres as bio-lubricants attenuate osteoarthritis. Small 16:e2004519. 32940012 10.1002/smll.202004519

[CIT0040] Yang Z, Bao Y, Chen W, He Y. (2020). Melatonin exerts neuroprotective effects by attenuating astro- and microgliosis and suppressing inflammatory response following spinal cord injury. Neuropeptides 79:102002. 31902595 10.1016/j.npep.2019.102002

[CIT0041] Yao C, Cao X, Yu B. (2021). Revascularization after traumatic spinal cord injury. Front Physiol 12:631500. 33995118 10.3389/fphys.2021.631500 PMC8119644

[CIT0042] Yuan XC, Wang P, Li HW, et al. (2017). Effects of melatonin on spinal cord injury-induced oxidative damage in mice testis. Andrologia 49:e12692. 10.1111/and.12692 27595881

[CIT0043] Zhai X, Ruan C, Ma Y, et al. (2018). 3D-bioprinted osteoblast-laden nanocomposite hydrogel constructs with induced microenvironments promote cell viability, differentiation, and osteogenesis both in vitro and in vivo. Adv Sci (Weinh) 5:1700550. 29593958 10.1002/advs.201700550 PMC5867050

[CIT0044] Zhang R, Xie L, Wu H, et al. (2020). Alginate/laponite hydrogel microspheres co-encapsulating dental pulp stem cells and VEGF for endodontic regeneration. Acta Biomater 113:305–16. 32663663 10.1016/j.actbio.2020.07.012

[CIT0045] Zhang W, Zhou G, Gao Y, et al. (2017). A sequential delivery system employing the synergism of EPO and NGF promotes sciatic nerve repair. Colloids Surf B Biointerfaces 159:327–36. 28806665 10.1016/j.colsurfb.2017.07.088

[CIT0046] Zhuang H, Bu S, Hua L, et al. (2016). Gelatin-methacrylamide gel loaded with microspheres to deliver GDNF in bilayer collagen conduit promoting sciatic nerve growth. Int J Nanomed 11:1383–94. 10.2147/IJN.S96324 PMC4824364 27099497

[CIT0047] Zou S, Wang B, Wang C, et al. (2020). Cell membrane-coated nanoparticles: research advances. Nanomedicine (Lond) 15:625–41. 32098564 10.2217/nnm-2019-0388

